# A retrospective cohort study of laparoscopic enhanced view totally extra-peritoneal Rives-Stoppa (eTEP-RS) repair of incisional ventral hernias in patients with morbid obesity

**DOI:** 10.1007/s00464-025-11565-z

**Published:** 2025-02-03

**Authors:** Shlomi Rayman, Mohamad Molham, Ran Orgad, Hana Gelman, Eliyahu Gorgov, Youri Mnouskin

**Affiliations:** 1 Department of General Surgery, Assuta Ashdod Public Hospital (Affiliated to the Faculty of Health and Science, Ben-Gurion University, Beer-Sheba, Israel), 7 Ha’Refua st., Ashdod, Israel; 2 The Azrieli Faculty of Medicine, Bar Ilan University, 8 Henrieta Szold st., Safed, Israel

**Keywords:** eTEP, Enhanced-view totally extra-peritoneal hernia repair, Rives-Stoppa, Laparoscopic ventral hernia repair, Incisional hernia, Morbid obesity

## Abstract

**Background:**

Incisional hernia (IH) repair in morbidly obese (MO) patients poses significant challenges due to higher risks of complications and recurrence. Traditional open repairs are linked to increased morbidity, driving interest in minimally invasive techniques. The enhanced view totally extra-peritoneal Rives-Stoppa (eTEP-RS) technique shows promise as a laparoscopic method for IH repair, but data on its efficacy and safety in MO patients are limited. This study aims to evaluate the efficacy, safety, and feasibility of the eTEP-RS approach specifically for IH repair in this high-risk population.

**Methods:**

Analysis of a retrospective cohort of consecutive patients undergoing laparoscopic eTEP-RS for IH repair between 2017 and 2022 which included 135 patients, categorized into two groups based on body mass index (BMI): the MO group (BMI > 35 kg/m^2^) and the control group (BMI ≤ 35 kg/m^2^). We compared demographics, comorbidities, hernia characteristics, intra-operative data, post-operative outcomes, and hernia recurrence rates.

**Results:**

Patients in the MO group had significantly more type 2 diabetes mellitus (*n* = 18, 51% vs *n* = 25, 25%; *p* = 0.004), hypertension (*n* = 35, 73% vs *n* = 75, 53%;* p* = 0.017), dyslipidemia (*n* = 29, 60% vs *n* = 58, 41%; *p* = 0.021), ASA score 3 (*n* = 18, 52% vs *n* = 23, 23%; *p* = 0.004), a history of previous umbilical hernia repair (*n* = 13, 27% vs *n* = 13, 9.2%; *p* = 0.002), and bariatric surgery (*n* = 10, 29% vs *n* = 13, 13%; *p* = 0.035). There were no differences in intra-operative characteristics, operative times, or intra-operative complications between groups. During a median follow-up period of 1 year (IQR 40–680 days), there were no differences in hernia recurrence (*n* = 2, 5.7% vs *n* = 9, 9%; *p* = 0.07), time to recurrence, or chronic analgesia usage between groups.

**Conclusion:**

The laparoscopic eTEP-RS approach was safe and effective for IH repair in patients with MO, demonstrating comparable post-operative outcomes and recurrence rates to those with a lower BMI in a selected cohort of patients.

**Supplementary Information:**

The online version contains supplementary material available at 10.1007/s00464-025-11565-z.

Morbid obesity (MO) is a continuous escalating global epidemic which is affecting an estimated 40% of adults worldwide with a subsequent impact on a wide range of health-related complications [1–3]. Among these, management of ventral incisional hernia (IH) represents a significant post-operative challenge, occurring in up to 35% of patients within three years of abdominal surgery and posing even higher risks in patients with MO [4–7]. The complexity of IH in patients with MO is exacerbated by impaired wound healing, increased intra-abdominal pressure, and difficulties associated with suturing a thickened abdominal wall during the initial operation. Notably, along with smoking, obesity represents a modifiable risk factor that significantly influences surgical outcomes [8–10].

These factors contribute to a high rate of hernia recurrence and post-operative morbidity, prompting recommendations for substantial pre-operative weight loss. While this approach may help in selecting excellent candidates for surgery, it often results in delays or prevents IH repairs in many patients with MO, thereby increasing their risk of hernia incarceration [6, 9, 11–13].

Over the past decade, the enhanced view totally extra-peritoneal Rives-Stoppa (eTEP-RS) IH repair has gained popularity as a prominent approach for the treatment of IH [14–19].

Despite the advancements in minimally invasive technology and its utilization, the literature on the application of laparoscopic eTEP-RS for IH repair in the MO patient population remains limited. As global MO rates continue to escalate, there is an increased demand for effective management strategies for IH in these patients and specifically the application of minimally invasive techniques. Recognizing the challenges of MO patients with IH, this study introduces a novel approach by examining the application of the eTEP-RS in this population—a demographic significantly underrepresented in abdominal wall surgery studies.

The aim of the current study was to examine the feasibility, safety, and efficacy of undertaking laparoscopic eTEP-RS for IH repair in patients with MO. Our hypothesis was that eTEP-RS for IH is safe, feasible, and has acceptable wound complications and hernia recurrence rates when utilized for patients with MO.

## Materials and methods

Following Institutional Review Board (IRB) approval, a prospectively maintained database of consecutive patients undergoing laparoscopic eTEP-RS for IH were retrospectively analyzed between November 2017 and December 2022 in Assuta Medical Center, University hospital, Ashdod, Israel. Inclusion criteria were adults over the age of 18 with a hernia at a previous incision site (IH). Exclusion criteria included patients under the age of 18, those with an infected mesh or ulceration of the abdominal wall, patients undergoing eTEP-RS for primary umbilical or epigastric ventral hernia repair, and patients undergoing ‘open’ or robotic repair. Patients who received chemotherapy within the year preceding surgery and those who underwent steroid therapy up to three months preoperatively were also excluded from this study. Subjects in our cohort were categorized into two groups based on Body Mass Index (BMI): Patients with a BMI > 35 kg/m^2^ (MO group) and patients with a BMI ≤ 35 kg/m^2^ (control group).

Pre-operative patient optimization included weight loss and smoking cessation recommendations, along with metabolic and bariatric surgery recommendation for those who were eligible.

Data retrieval included demographics, anthropometrics, comorbidities, the American Society of Anesthesiologists (ASA) score, previous surgical history, hernia size, type of mesh used, post-operative course, hernia recurrence, and need for secondary surgery. All patients underwent a pre-operative evaluation by a senior abdominal wall surgeon and an abdomen/pelvic CT scan for hernia classification. Hernia size and location were classified according to the European Hernia Society (EHS) classification [20]. Post-operative complications were graded by the Clavien–Dindo classification (CD) [21] and major complications were considered at CD ≥ 3. All operations were undertaken by the same abdominal wall surgery team lead by the head of the abdominal wall surgery unit. Post-operative care included early mobilization, initiation of full liquid diet, routine analgesic treatment, and venous thrombosis prophylaxis with low-molecular weight heparin and/or lower limb sequential compression devices. Patients discharged following return to normal diet, pain controlled by oral medication, and full mobilization. Patients were instructed to avoid heavy lifting over 5 kg up to 6 weeks post-operatively.

Patients were routinely evaluated by their surgeons or other surgical attendings at our out-patient clinic, 3–5 weeks after surgery, and then yearly. The primary outcome evaluated was hernia recurrence, defined as disruption of the previously repaired anterior rectus fascia and confirmed via physical exam and CT scan when suspected. Secondary outcomes included peri-operative complications and chronic pain. Patients operated semi-electively were those who were admitted and referred to surgery via the emergency department. The surgical technique was implemented as described by Radu et al. [14].

This study was reported in accordance with the STROCSS guidelines and received ethical approval from the IRB Committee No. AAA-0046-22 and in accordance with the Declaration of Helsinki. No informed consent was required to undertake this study.

### Statistical analysis

Descriptive results were presented as a number and percentage for categorical variables and as a mean ± standard deviation (SD) or median + inter quarterly range (IQR) for continuous variables. Categorical variables were compared using Chi-square tests or Fisher’s exact test, and continuous variables were compared using *t* test or Wilcoxon rank sum test according to distribution of data samples. A *p* value of < 0.05 was considered statistically significant. Statistical analyses, figures, and graphs were produced with R Core Team (2024). R: A Language and Environment for Statistical Computing. R Foundation for Statistical Computing, Vienna, Austria. <https://www.R-project.org/ >

## Results

During the study period, 135 patients underwent eTEP-RS for IH with a mean age of 64.1 ± 12 and a gender distribution of 82 females (61%) and 53 males (39%). Of them, 35 patients had a BMI > 35 kg/m^2^ (MO group) and 100 patients had a BMI ≤ 35 kg/m^2^ (control). Patients in the MO group had significantly more type 2 diabetes mellitus (*n* = 18, 51% vs *n* = 25, 25%; *p* = 0.004), hypertension (*n* = 35, 73% vs *n* = 75, 53%;* p* = 0.017), dyslipidemia (*n* = 29, 60% vs *n* = 58, 41%; *p* = 0.021), ASA score 3 (*n* = 18, 52% vs *n* = 23, 23%; *p* = 0.004), a history of previous umbilical hernia repair (*n* = 13, 27% vs *n* = 13, 9.2%; *p* = 0.002), and bariatric surgery (*n* = 10, 29% vs *n* = 13, 13%; *p* = 0.035). The MO group had significantly less smokers (*n* = 1, 2.9% vs *n* = 22, 22%; *p* = 0.01) and less previous colon surgery (*n* = 1, 2.9% vs *n* = 19, 19%; *p* = 0.021; Table 1).

**Table 1:** Patient’s demographics, past medical, and surgical history

Characteristic	Overall, *N* = 135^a^	BMI > 35, * N* = 35^a^	BMI ≤ 35, * N* = 100^a^	*p* value^b^
Age at surgery	64.1 (12.0)	63.9 (10.3)	64.2 (12.6)	0.9
Gender				0.057
Female	82 (61%)	26 (74%)	56 (56%)	
Male	53 (39%)	9 (26%)	44 (44%)	
BMI	31.5 (6.0)	39.2 (4.6)	28.8 (3.6)	** < 0.001**
ASA score				**0.004**
1	6 (4.4%)	0 (0%)	6 (6.0%)	
2	86 (64%)	16 (46%)	70 (70%)	
3	41 (30%)	18 (51%)	23 (23%)	
4	2 (1.5%)	1 (2.9%)	1 (1.0%)	
IHD	19 (14%)	4 (11%)	15 (15%)	0.8
DM	43 (32%)	18 (51%)	25 (25%)	**0.004**
Hypertension	87 (64%)	28 (80%)	59 (59%)	**0.025**
Dyslipidemia	64 (47%)	22 (63%)	42 (42%)	**0.033**
COPD	11 (8.1%)	3 (8.6%)	8 (8.0%)	> 0.9
History of smoking	23 (17%)	1 (2.9%)	22 (22%)	**0.010**
Smoking Pack Years	40.4 (46.0)	30.0 (NA)	40.8 (46.9)	0.9
Anti-coagulation Tx	37 (27%)	8 (23%)	29 (29%)	0.5
Previous surgery				
Gynecological	43 (32%)	13 (37%)	30 (30%)	0.4
General	107 (79%)	30 (86%)	77 (77%)	0.3
Urological	16 (12%)	3 (8.6%)	13 (13%)	0.8
Colon resection	20 (15%)	1 (2.9%)	19 (19%)	**0.021**
Cholecystectomy	37 (27%)	11 (31%)	26 (26%)	0.5
Bariatric	23 (17%)	10 (29%)	13 (13%)	**0.035**
Laparoscopic surgery	65 (48%)	20 (57%)	45 (45%)	0.2
Ventral hernia repair	21 (16%)	9 (26%)	12 (12%)	0.054
Umbilical hernia repair	25 (19%)	12 (34%)	13 (13%)	**0.005**
Inguinal hernia repair	9 (6.7%)	2 (5.7%)	7 (7.0%)	> 0.9
Location of abdominal scar				
Midline	86 (64%)	21 (60%)	65 (65%)	0.6
Para-median	7 (5.2%)	1 (2.9%)	6 (6.0%)	0.7
Port-site	32 (24%)	8 (23%)	24 (24%)	0.9
Kocher	7 (5.2%)	2 (5.7%)	5 (5.0%)	> 0.9
Transverse	7 (5.2%)	2 (5.7%)	5 (5.0%)	> 0.9
Pfannenstiel	17 (13%)	5 (14%)	12 (12%)	0.8

Bold values indicate statistical significance (*p* < 0.05)

*BMI* body mass index, *ASA* American society of anesthesiologists, *IHD* ischemic heart disease, *DM* diabetes mellitus, *COPD* chronic obstructive pulmonary disease, *Tx* treatment

^a^Mean (SD); *n* (%)

^b^Welch Two-Sample *t* test; Pearson’s Chi-squared test; Wilcoxon rank sum test; and Fisher’s exact test

**Fig. 1:**
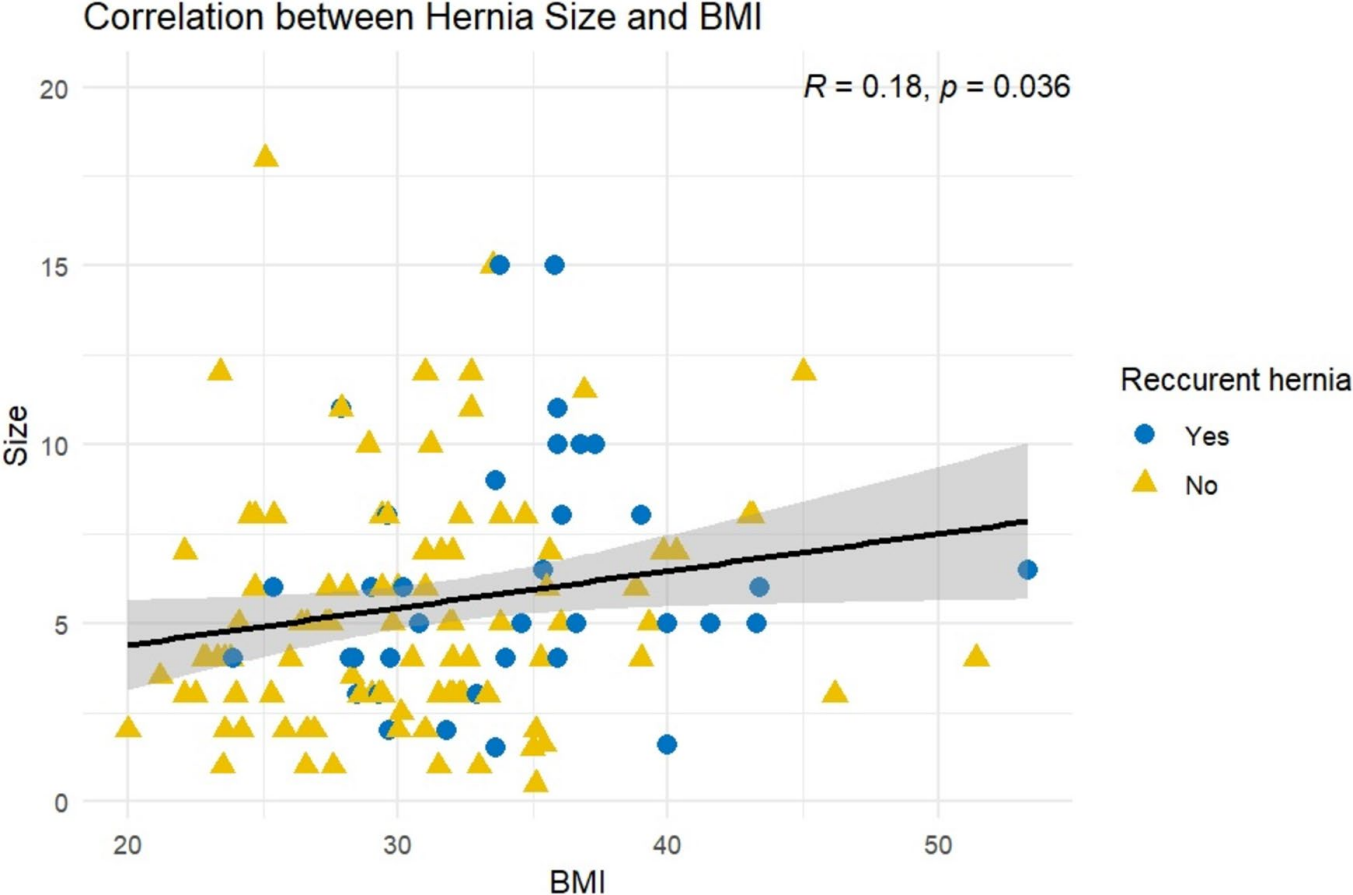
Scatter plot representing the correlation between hernia size and body mass index

There were 46 patients (35%) who had previously undergone umbilical or ventral hernia repair with significantly more patients in the MO group undergoing eTEP-RS for a recurrent IH (*n* = 17, 49% vs *n* = 20, 20%; *p* = 0.001). Furthermore, patients in the MO group had significantly larger width (W2) defects according to the EHS classification (*n* = 21. 60% vs *n* = 35, 35%; *p* < 0.001; Table 2). There was a significantly positive correlation between BMI and hernia size measured by CT scan (*R* = 0.18, *p* = 0.036; Fig. 1). Our study included 44 patients (12 in the MO group) who underwent such an approach of eTEP-TAR, which 13 of them (6 in MO group, 7 in control group) had a W3 defect. There was no difference in hernia recurrence groups regarding patients undergoing TAR (*n* = 1 vs *n* = 2; *p* > 0.9; data not shown).

**Table 2:** European Hernia Society—Incisional Hernia classification

Characteristic	Overall, * N* = 135^a^	BMI > 35, * N* = 35^a^	BMI ≤ 35, * N* = 100^a^	*p* value^b^
Midline				
M1 (subxiphoid)	18 (13%)	6 (17%)	12 (12%)	0.6
M2 (epigastric)	74 (55%)	22 (63%)	52 (52%)	0.3
M3 (umbilical)	102 (76%)	29 (83%)	73 (73%)	0.2
M4 (infraumbilical)	36 (27%)	10 (29%)	26 (26%)	0.8
M5 (suprapubic)	14 (10%)	4 (11%)	10 (10%)	0.8
Lateral				
L1 (subcostal)	3 (2.2%)	1 (2.9%)	2 (2.0%)	> 0.9
L2 (flank)	8 (5.9%)	1 (2.9%)	7 (7.0%)	0.7
L3 (iliac)	3 (2.2%)	0 (0%)	3 (3.0%)	0.6
L4 (lumbar)	1 (0.7%)	0 (0%)	1 (1.0%)	> 0.9
Width				** < 0.001**
W1 (< 4 cm)	59 (44%)	5 (14%)	54 (54%)	
W2 (4-10 cm)	56 (41%)	21 (60%)	35 (35%)	
W3 (> 10 cm)	20 (15%)	9 (26%)	11 (11%)	
Recurrent hernia	37 (27%)	17 (49%)	20 (20%)	**0.001**
Concomitant midline hernias	71 (53%)	22 (63%)	49 (49%)	0.2
Concomitant lateral hernias	2 (1.5%)	0 (0%)	2 (2.0%)	> 0.9
Concomitant midline and lateral hernias	6 (4.4%)	1 (2.9%)	5 (5.0%)	> 0.9

Bold values indicate statistical significance (*p* < 0.05)

^a^*n* (%)

^b^Fisher’s exact test; Pearson’s Chi-squared test

Intra-operative characteristics such as the urgency, transversus abdominis release (TAR), diastasis recti repair, concomitant inguinal hernia repair, and mesh type and size were similar between groups, with no significant differences in operative times or intra-operative complication (Table 3). There were no significant differences in hospital opioid and NSAID’s treatment, length of hospital stay, post-operative complications, CD scores, or other specific post-operative events, such as seroma, wound infection, and ileus between the groups (Table 4). During a median follow-up period of 1 year (IQR 40–680 days), we found 11 cases of recurrence (10 cases identified by physical examination and CT scan and 1 case by physical examination alone). There were no differences in hernia recurrence (*n* = 2, 5.7% vs *n* = 9, 9%; *p* = 0. 7), time to recurrence, or chronic analgesia usage between groups (Table 5). For 29 patients, long-term (> 2 years) follow-up data were available. In these patients, the median follow-up was 1022 days and we found recurrence in a total of 4 patients (2 in each group, *p* > 0.9). Of note, significantly fewer patients in MO group had post-operative CT scans (*n* = 11, 31% vs *n* = 51, 51%, *p* = 0.046).

**Table 3:** Hernia—intra-operative characteristics

Characteristic	Overall, * N* = 135^a^	BMI > 35, * N* = 35^a^	BMI ≤ 35, * N* = 100^a^	*p* value^b^
Urgency				0.3
Elective	125 (93%)	31 (89%)	94 (94%)	
Semi-elective	10 (7.4%)	4 (11%)	6 (6.0%)	
Transversus abdominis release				
Right	28 (21%)	9 (26%)	19 (19%)	0.4
Left	6 (4.4%)	1 (2.9%)	5 (5.0%)	> 0.9
Bilateral	10 (7.4%)	2 (5.7%)	8 (8.0%)	> 0.9
Diastasis recti repair	42 (31%)	9 (26%)	33 (33%)	0.4
Concomitant inguinal hernia repair	36 (27%)	5 (14%)	31 (31%)	0.054
Mesh type				> 0.9
Non-absorbable	55 (41%)	15 (43%)	40 (40%)	
Partially absorbable	70 (52%)	18 (51%)	52 (52%)	
Composite	10 (7.4%)	2 (5.7%)	8 (8.0%)	
Mesh size				0.2
< 15 cm	4 (3.0%)	0 (0%)	4 (4.0%)	
15-30 cm	22 (16%)	3 (8.6%)	19 (19%)	
> 30 cm	109 (81%)	32 (91%)	77 (77%)	
Mesh fixation	38 (28%)	6 (17%)	32 (32%)	0.093
Use of drain	15 (11%)	5 (14%)	10 (10%)	0.5
Intra-op bleeding	6 (4.4%)	1 (2.9%)	5 (5.0%)	> 0.9
PBC during op	3 (2.2%)	1 (2.9%)	2 (2.0%)	> 0.9
Operative time (minutes)	167.3 (66.0)	179.5 (76.3)	163.0 (61.9)	0.3
Intra-op complications	5 (3.7%)	2 (5.7%)	3 (3.0%)	0.6

PBC- Packed red blood cells

^a^*n* (%); mean (SD)

^b^Fisher’s exact test; Pearson’s Chi-squared test; and Wilcoxon rank sum test

**Table 4:** Hernia—post-operative course

Characteristic	Overall, * N* = 135^a^	BMI > 35, * N* = 35^a^	BMI ≤ 35, * N* = 100^a^	*p* value^b^
Hospital Opioid Tx	61 (45%)	14 (40%)	47 (47%)	0.5
Hospital NSAIDS Tx	29 (21%)	8 (23%)	21 (21%)	0.8
Hospital stay (days)	2.5 (1.6)	2.7 (1.2)	2.5 (1.7)	0.14
Post-operative complications	22 (16%)	6 (17%)	16 (16%)	0.9
Clavien–Dindo classification				> 0.9
1	13 (59%)	4 (67%)	9 (56%)	
2	2 (9.1%)	0 (0%)	2 (13%)	
3	7 (32%)	2 (33%)	5 (31%)	
Seroma	17 (13%)	5 (14%)	12 (12%)	0.8
Wound infection	6 (4.4%)	2 (5.7%)	4 (4.0%)	0.6
Ileus	2 (1.5%)	0 (0%)	2 (2.0%)	> 0.9
Small bowel obstruction	2 (1.5%)	0 (0%)	2 (2.0%)	> 0.9
Rectus sheath dehiscence				
Posterior	13 (9.6%)	5 (14%)	8 (8.0%)	0.3
Anterior	2 (1.5%)	0 (0%)	2 (2.0%)	> 0.9
Post-operative antibiotics	6 (4.4%)	2 (5.7%)	4 (4.0%)	0.6
Drainage (IR)	4 (3.0%)	2 (5.7%)	2 (2.0%)	0.3
Re-operation	5 (3.7%)	1 (2.9%)	4 (4.0%)	> 0.9
Mesh removal	4 (3.0%)	2 (5.7%)	2 (2.0%)	0.3
Bowel resection	2 (1.5%)	1 (2.9%)	1 (1.0%)	0.5
Maximal VAS score	2.8 (2.6)	2.6 (2.6)	2.8 (2.6)	0.7

*Tx* treatment, *IR* invasive radiology, *VAS* visual analog scale

^a^*n* (%); mean (SD)

^b^Pearson’s Chi-squared test; Wilcoxon rank sum test; and Fisher’s exact test

**Table 5:** Hernia—follow-up

Characteristic	Overall, * N* = 135^a^	BMI > 35, * N* = 35^a^	BMI ≤ 35, * N* = 100^a^	*p* value^b^
Median follow-up, days (IQR)	364.5 (40.0,680.5)	520.0 (45.0,954.5)	290.0 (41.0,639.5)	0.2
Hernia recurrence	11 (8.1%)	2 (5.7%)	9 (9.0%)	0.7
Time to recurrence (months)	1.2 (6.7)	0.7 (4.1)	1.4 (7.4)	0.5
Chronic analgesic Tx	2 (1.5%)	1 (2.9%)	1 (1.0%)	0.5
Other abdominal surgery after hernia repair	8 (5.9%)	1 (2.9%)	7 (7.0%)	0.7
Post-op CT scan	62 (46%)	11 (31%)	51 (51%)	**0.046**

Bold values indicate statistical significance (*p* < 0.05)

*IQR* interquartile range, *Tx* treatment

^a^Median (25%,75%); *n* (%); mean (SD)

^b^Wilcoxon rank sum test; Fisher’s exact test; and Pearson’s Chi-squared test

## Discussion

MO poses a dual challenge in abdominal wall reconstructive surgery, significantly increasing the risk of IH occurrence as well as the likelihood of hernia recurrence and wound complications post-repair [22]. This is the first study examining the safety, feasibility, post-operative complications, and hernia recurrence following laparoscopic eTEP-RS in this high-risk patient population. Our findings highlighted that despite the increased presence of obesity-related comorbidities, such as diabetes and hypertension, and a higher incidence of patients with larger hernia widths and a history of previous hernia repairs in the morbidly obese group, there were no significant differences in post-operative morbidity or hernia recurrence rates when compared to patients with a BMI of equal or less than 35 kg/m^2^. This finding is particularly notable given the extended follow-up period, emphasizing the effectiveness of the laparoscopic eTEP-RS approach in managing IH in patients with MO.

Despite the varieties of surgical techniques available for abdominal wall reconstruction, the literature on IH repair in patients with MO remains scarce, particularly regarding advanced minimally invasive techniques such as the eTEP-RS approach. Additionally, the limited amount of randomized controlled trials on the topic makes it challenging to formulate guidelines and determine the superiority of one technique over another, with most publications being retrospective. The PROVE-IT trial by Petro et al., randomized 75 patients with ventral hernias (primary and incisional) to either laparoscopic (*n* = 36) to robotic (*n* = 39) ventral hernia repair (intra-peritoneal onlay mesh; IPOM) with significantly higher BMI of ≥ 35 kg/m^2^ in the robotic group. The authors showed no difference in post-operative complications between groups [23]. A retrospective trial of 322 patients examining the efficacy of IPOM in patients with MO by Maspero et al. found that patients (*n* = 36) with class III obesity (BMI ≥ 40 kg/m^2^) who underwent IPOM had significantly longer hospital stays compared to patients with class I and II obesity, but without differences in post-operative complications or hernia recurrence at 1-year follow-up [24]. As for advanced minimally invasive techniques, Belyansky et al. described the use of a robotic approach in a cohort of 37 patients with a mean BMI of 35.5 kg/m^2^, which included 15 patients with an IH. The authors reported minimal post-operative complications with no early recurrences over a short period of follow-up of 36 days [17]. Similarly, Baig et al. formulated an algorithm based on a retrospective analysis of 50 patients directed at optimal hernia repair according to favorable and unfavorable obesity and hernia characteristics. Of the techniques used, endoscopic Rives-Stoppa and endoscopic TAR were utilized in 2 and 9 patients with a mean BMI of 32.3 ± 1.5 and 33.7 ± 2.9 kg/m^2^, respectively. At a mean follow-up period of 17.6 ± 7.2 months, the authors report of 2 recurrences of patients who underwent emergent operations [11]. Our study echoes these findings that despite increased obesity-related comorbidities and more complex hernia presentations among patients with MO, no significant differences in post-operative morbidity or hernia recurrence rates were observed when compared to the control group. Conversely, Tsereteli et al., retrospectively reviewed 1071 patients who underwent IPOM with trans-facial sutures fixation over a 13-year period. The study included both primary and incisional hernias, and found 134 with a BMI over 40 kg/m^2^. Along with increased operative time and hospital stay, the authors report a significantly higher rate of hernia recurrence in patients with MO (8.3% vs 2.9%; p < 0.001) at a median follow-up of 19 months [25]. Additionally, the minimal post-operative complications reported by Belyansky et al. [17] in their early experiences with robotic eTEP-RS are consistent with our findings, suggesting that the eTEP-RS method, whether robotic or laparoscopic, may be particularly suited to managing the complexities associated with MO. Similarly, the algorithm proposed by Baig et al. [11], which tailors hernia repair techniques based on patient-specific obesity and hernia characteristics, aligns with our practice of careful pre-operative planning and patient selection to optimize surgical outcomes. Our study underscores the adaptability of this surgical technique to effectively handle the complexities introduced by larger hernia sizes and prior surgical interventions, further reinforcing its value in a clinical setting where MO is a growing concern and challenges surgeons on a daily basis. It is our opinion that the advantages of the eTEP-RS over other techniques, aside from its minimally invasive approach which is associated with early recovery, is the ability to perform a retro-muscular reinforcement of the entire abdominal wall, without a midline incision above a previous scar with enlargement of the defect over viable tissue. This preservation of viable midline skin and subcutaneous tissue is what may offer the increased strength of the eTEP-RS approach. The eTEP technique allows a versatile approach to complex abdominal wall reconstruction, developing an extensive retro-muscular plane with the option to extend the operation to include TAR in situations that necessitate further posterior component separation, such as W3 hernias. Furthermore, this study demonstrates the applicability and feasibility of undertaking eTEP-TAR in patients with morbid obesity, highlighting its potential benefits in managing this challenging patient population. While MO is considered a modifiable pre-operative risk factor for abdominal wall reconstruction, many surgeons hesitate to repair IH in patients with MO, often recommending an initial weight loss before they may qualify for surgical repair. This practice is problematic as the concomitant presence of MO and IH significantly limits patients’ ability to engage in physical activity, thus exacerbating the very issue of weight loss that is crucial for their eligibility for hernia repair. Furthermore, the ability of patients with MO to lose a significant amount of weight without medical or surgical intervention is limited, leading to additional delays in abdominal wall reconstruction and increasing the likelihood of hernia-related morbidity.

A pivotal highlight of this study is the successful utilization of the laparoscopic approach to perform eTEP-RS repairs, a method easily facilitated by robotic platforms when and where available. In settings like ours, where robotic systems are not readily accessible, the need to excel in advanced laparoscopic techniques becomes paramount. Our study demonstrates that even without the advanced capabilities of a robotic platform, significant surgical advancements can be achieved in managing complex abdominal wall reconstructions in patients with MO. Hence, it is our opinion that the eTEP-RS technique, in simple or complex situations, may be reproduced by minimally invasive surgeons globally, especially by those who may not have access to a robotic platform. It underscores the potential for widespread adoption of this approach, ensuring that high-quality surgical care is accessible regardless of local resources. This adaptation is crucial not only for widening the accessibility of high-level surgical care worldwide but also for ensuring that patients in diverse global settings benefit from minimally invasive surgical innovations. Another advantageous characteristic of patients with MO undergoing eTEP-RS is the enlarged rectus abdominis muscles. These larger muscles provide increased space for surgical maneuvers during dissection and facilitate the reinforcement of abdominal wall reconstruction. The expanded muscle size allows for the placement of larger mesh, covering more surface area, which can enhance the overall stability and effectiveness of the hernia repair.

The strength of our study is the focus on a difficult study group which is often deferred from operative management due to a concern regarding peri-operative complications and outcomes. In addition, the operative technique is uniform in our cohort which allows accurate comparison of surgical outcomes between groups. To our knowledge, this is the largest study cohort regarding patients with MO undergoing laparoscopic eTEP-RS.

Limitations of the study are inherent to its design and methodology which include a retrospective analysis, small sample size, and short-medium follow-up. There is also a selection bias of patients with MO which may have been excluded in situations where they seemed unfit to undergo a major abdominal operation during their clinic examination. We also found a significantly lower percentages of MO patients that had post-operative CT scans and this may influence identification of hernia recurrence. Since CT scans were only performed if indicated by the clinical situation, we cannot fully control for this possible confounding factor. Long-term follow-up data (> 2 years) was only available for a small subset of patients (21%), and although we found no difference in hernia recurrence rate, further long-term studies are required to confirm this. Despite these limitations, our study offers a valuable addition to the literature regarding the approach to IH in patients with morbid obesity. Taken together, our results suggest that previous concerns regarding peri-operative complications and outcomes in patients with MO undergoing IH repair are not applicable to laparoscopic eTEP-RS repairs, which point to this technique as a viable approach for IH repair in this patient population.

## Conclusion

The laparoscopic eTEP-RS approach was safe and effective for IH repair in patients with MO, demonstrating comparable post-operative outcomes and recurrence rates to those with a lower BMI in a selected cohort of patients.

## Supplementary Information

Below is the link to the electronic supplementary material.

Supplementary file1 (DOCX 31 kb)
